# *Chlamydia psittaci* comparative genomics reveals intraspecies variations in the putative outer membrane and type III secretion system genes

**DOI:** 10.1099/mic.0.000097

**Published:** 2015-07

**Authors:** Bernard J. Wolff, Shatavia S. Morrison, Denise Pesti, Satishkumar Ranganathan Ganakammal, Ganesh Srinivasamoorthy, Shankar Changayil, M. Ryan Weil, Duncan MacCannell, Lori Rowe, Michael Frace, Branson W. Ritchie, Deborah Dean, Jonas M. Winchell

**Affiliations:** ^1^​ Infectious Diseases Laboratory, College of Veterinary Medicine, University of Georgia, Athens, GA, USA; ^2^​ Respiratory Diseases Branch, Centers for Disease Control and Prevention, Atlanta, GA, USA; ^3^​ The National Center for Emerging and Zoonotic Infectious Diseases Branch, Centers for Disease Control and Prevention, Atlanta, GA, USA; ^4^​ Children's Hospital Oakland Research Institute, Oakland, CA; ^5^​ UCSF and UC Berkeley Joint Graduate Program in Bioengineering, Oakland, CA

## Abstract

*Chlamydia psittaci* is an obligate intracellular bacterium that can cause significant disease among a broad range of hosts. In humans, this organism may cause psittacosis, a respiratory disease that can spread to involve multiple organs, and in rare untreated cases may be fatal. There are ten known genotypes based on sequencing the major outer-membrane protein gene, *ompA*, of *C. psittaci.* Each genotype has overlapping host preferences and virulence characteristics. Recent studies have compared *C. psittaci* among other members of the *Chlamydiaceae* family and showed that this species frequently switches hosts and has undergone multiple genomic rearrangements. In this study, we sequenced five genomes of *C. psittaci* strains representing four genotypes, A, B, D and E. Due to the known association of the type III secretion system (T3SS) and polymorphic outer-membrane proteins (Pmps) with host tropism and virulence potential, we performed a comparative analysis of these elements among these five strains along with a representative genome from each of the remaining six genotypes previously sequenced. We found significant genetic variation in the Pmps and tbl3SS genes that may partially explain differences noted in *C. psittaci* host infection and disease.

## Introduction

The *Chlamydiaceae* family of bacteria comprises nine distinct species, namely *Chlamydia trachomatis*, *C. suis*, *C. muridarum*, *C. psittaci*, *C. pneumoniae*, *C. abortus*, *C. felis*, *C. pecorum* and *C. caviae* (Read *et al.*, 2013). This diverse group of obligate intracellular Gram-negative bacteria have adapted to their host cell niche by evolving to use a complex biphasic life cycle, which consists of a metabolically inactive, infectious form known as an elementary body (EB) and a metabolically active and non-infectious reticulate body (RB) (Abdelrahman & Belland, 2005). *Chlamydiaceae* infect a broad range of hosts, and evidence of infection has been found in nearly every phylogenetic group of animals (Kaleta & Taday, 2003; Read *et al.*, 2003).

*C. psittaci* is primarily a zoonotic pathogen that is normally transmitted through close contact with infected birds and some mammals including cattle, pigs, sheep, swine, goats, cats and horses, not to mention feral animals (Hotzel *et al.*, 2004; Read *et al.*, 2013). The bacteria are often found in secretions and faecal droppings, remaining infectious for up to 30 days (Beeckman & Vanrompay, 2009; Haag-Wackernagel & Moch, 2004; Harkinezhad *et al.*, 2009; Heddema *et al.*, 2006a). Infection in humans may lead to psittacosis, a severe respiratory illness often associated with multi-organ involvement causing significant morbidity and mortality (Smith *et al.*, 2011). *C. psittaci* is also a major cause of economic loss in the poultry industry in the US and abroad, and poses a significant risk to farm workers as well as having potential for laboratory-acquired infection (Gaede *et al.*, 2008; Miller *et al.*, 1987; Smith *et al.*, 2011). Because of the potential public health risk of *C. psittaci*, the National Association of State Public Health Veterinarians published a compendium on testing and strategies for managing the disease in birds and humans (Smith *et al.*, 2011).

*C. psittaci* has the widest documented host range within the *Chlamydiaceae* family including avian, mammalian, reptilian and human hosts (Harkinezhad *et al.*, 2009). *C. psittaci* is classified into ten genotypes, designated A–G, E/B, M56 and WC (Read *et al.*, 2013; Van Lent *et al.*, 2012). Recently, a real-time PCR assay targeting differences in the *ompA* gene was developed to differentiate the genotypes (Mitchell et al., 2009). Biological differences in host preference and virulence can be noted between the different *C. psittaci* genotypes. For instance, genotype A is endemic in psittacine birds and is theorized to be a common cause of respiratory disease and/or flu-like symptoms in exposed humans, while genotypes C and D have primarily been associated with waterfowl and poultry, respectively (Heddema *et al.*, 2006a, c; Smith *et al.*, 2011). Genotype E has been shown to infect a diverse group of avian species, including psttacines, pigeons, waterfowl and turkeys, and was first described to infect humans in the late 1920s following exposure to diseased parrots (Harkinezhad *et al.*, 2007). Although all genotypes may infect humans, including genotypes from both avian and mammalian species, genotype A has been referenced as the most common cause of human disease. There is substantial variation in the likelihood of infection and disease following exposure to infected birds, even within what is currently classified as the same genotype (Heddema *et al.*, 2006b, c; Wreghitt & Taylor, 1988).

Genetic manipulation techniques cannot reliably be used on *Chlamydiaceae* due to their intracellular requirement for replication. As a result, research on the specific interactions and contributions of genes to virulence, infectivity and replication are limited. Comparisons between the various genotypes and between species are limited despite some recent advancements with better characterized species and the use of surrogate systems (Peters *et al.*, 2007). While some members of the *Chlamydiaceae* family have been sequenced and studied for their host and tissue preferences and virulence mechanisms, only 16 have been completely characterized to date (Kalman *et al.*, 1999; Read *et al.*, 2000, 2003, 2013; Stephens *et al.*, 1998; Thomson *et al.*, 2005; Van Lent *et al.*, 2012; Voigt *et al.*, 2012). Next generation sequencing bypasses some of the limitations of bench work by allowing more meaningful investigation of the genotypes at the genomic level and their potential association with host specificity and virulence.

Here, we report on the comparative genomics of all ten genotypes of *C. psittaci* of which genotypes A, B, D and E were sequenced in this study. Our research focused on the genes encoding polymorphic membrane proteins (Pmps) and the type III secretion system (T3SS) proteins because of their known involvement in pathogenicity (Voigt *et al.*, 2012). The Pmps are a large family of proteins unique to *Chlamydiaceae*, which are highly variable in numbers and homology among the family, and are thought to be involved in niche adaptation based on adherence to the host cell, molecular transport and cell wall associated functions (Rockey *et al.*, 2000). The tbl3SS transports effector proteins into the host cytoplasm using a needle-like apparatus, similar to other Gram-negative bacteria (Hueck, 1998). While the structural genes are well conserved, the secreted effector proteins, although difficult to identify, are widely diverse and have many unique functions (Valdivia, 2008). Some secreted effectors have been identified and fairly well characterized. Specifically, the *tarp* gene in *C. trachomatis* was well characterized by Somboonna *et al.* (2011) as a virulence factor and has been associated with actin recruitment and inclusion development. We found numerous insertions, deletions and single nucleotide polymorphisms among the *pmp* and tbl3SS genes that may account for host preferences and virulence characteristics for the *C. psittaci* genotypes.

## Methods

### *C. psittaci* strains

*C. psittaci* strains DD-34 (ATCC VR-854, genotype A), CP3 (ATCC VR-574, genotype B), NJ1 (genotype D), Frances (ATCC VR-122, genotype E) and a genotype A strain (UGA) recovered from a cockatiel, were sequenced in this study. The DD-34 strain was originally isolated from a parrot in 1949 (Davis, 1949). The CP3 strain was isolated from a pigeon in 1958 (Page, 1966). The Frances strain was isolated from a ferret inoculated with human material in 1934 (Francis & Magill, 1938 *)*. *C. psittaci* CP3 and NJ1 genomic sequences are also available elsewhere (Van Lent *et al.*, 2012), while Frances, DD-34 and UGA are newly described in the current study. This study represents comprehensive analysis of these genomes using the whole genome assembly and annotation methods describe below.

### *C. psittaci* culture

*C. psittaci* culture was performed as previously described (Mitchell et al., 2009). Briefly, *C. psittaci* reference strains were propagated in Vero cell monolayers grown in 150 cm^2^ culture flasks in Eagle's minimum essential medium (MEM) supplemented with MEM nonessential amino acids, 2 μM l-glutamine, 20 μM HEPES buffer, 10 % FCS, 20 μg streptomycin ml^− 1^ and 25 μg vancomycin ml^− 1^. Confluent cell monolayers were inoculated by replacing the growth medium with 5 ml of stock *C. psittaci* culture diluted 1:10 in MEM containing 1 μg cycloheximide ml^− 1^. The inoculated monolayers were placed at 37 °C and 5 % CO_2_ for 2 h before an additional 50 ml of MEM containing cycloheximide was added to each flask. Cultures were incubated for 7 days at 37 °C or until the monolayers demonstrated nearly 100 % cytopathic effects. The cell culture was transferred to 50 ml tubes and stored at − 80 °C prior to EB isolation.

EB isolation was performed by density-gradient centrifugation as previously described (Mukhopadhyay *et al.*, 2004). DNA was extracted from the pellet using a QiaAmp DNA minikit (Qiagen) according to the manufacturer's instructions and frozen at − 80 °C prior to sequencing.

### Whole genome sequencing and assembly

The genomic DNA for *C. psittaci* strains DD-34, CP3, NJ1, Frances and UGA was prepared for whole genome paired-end sequencing on an Illumina GAIIx DNA sequencer using standard protocols and reagents from Illumina. Approximately 1 μg of genomic DNA was sheared using a Covaris S2 sonicator (Covaris) to a mean size of 350 bp. DNA sequencing libraries were then prepared using Illumina Truseq chemistry and size selected using double Ampure (Beckman Coulter) selection. Paired-end flowcells underwent cluster formation using an Illumina cBot, followed by 100 × 100 bp cycle sequencing using SBS cycle sequencing V5 kits. Sequence data were processed using casava (v1.8.2) into paired fastq read sets. Read quality checks were performed using a combination of publicly available tools and in-house scripts. *C. psittaci* 6BC (NC_015740) was used as reference genome for all analysis. Trimming of reads based on quality, mapping of reads to a reference genome, and *de novo* assembly were performed using CLC Genomics Workbench 5.5.1. Since CLC Genomics 5.5.1 was no longer supported on the computing environment, CLC Genomics Workbench 7.0.4 was used to assembly the RTH sequence reads.

### Whole genome annotation

All five genomes were submitted to NCBI and annotated using the NCBI Prokaryotic Genomes Automatic Annotation Pipeline (PGAAP) (http://www.ncbi.nlm.nih.gov/genomes/static/Pipeline.html). The GenBank accession numbers for each genome are listed in Table 1.

**Table 1:** Whole genome sequencing and annotation results

	Strain				
Characteristic	DD34	UGA	CP3	NJ1	Frances
Genotype	A	A	B	D	E
GenBank accession no.	AFVL00000000	AWXQ00000000	AFVN00000000	AFVK00000000	AFVM00000000
Total assembled size (bp)	1 163 748	1 164 948	1 163 075	1 160 660	1 162 120
Plasmid size (bp)	7553	7553	7553	7532	7545
Total reads	13 181 088	12 562 928	12 695 762	7 803 252	13 291 438
Total bases	1 317 642 394	1 256 351 619	1 268 662 067	780 739 971	1 327 907 989
Mean read length	101	101	101	101	101
Mean coverage	576.2	548	556.1	340.4	581.1
Number of contigs	4	6	4	3	2
N50	778 174	779 321	778 149	775 399	1 159 687
Genes predicted	1057	1059	1054	1057	1044
tRNAs predicted	38	38	38	38	38
DNA G+C content (mol%)	39.03	39.02	39.01	38.95	39.03

### Whole genome comparison

The *de novo* assembled contigs for each sample were ordered against the 6BC reference genome using abacas tool (Assefa *et al.*, 2009). The ordered samples' superscaffolded genomes and reference genome were compared using Mauve to identify conserved and rearranged regions (Darling *et al.*, 2010). The above input was used for Blast Ring Image Generator (BRIG) analysis to identify differences (Alikhan *et al.*, 2011). Using the whole genome SNPs, identified by the kSNP version 2.1.2 (Gardner & Hall, 2013) software application, a maximum-likelihood phylogenetic tree was reconstructed with the RAxML version 7.3.0-pthread (Stamatakis, 2014). The tree was reconstructed with 1000 replicates for bootstrapping (Gardner & Hall, 2013). The tree was visualized with Figtree (Rambaut, 2015). The phylogenetic tree represents the evolutionary relationship of *C. psittaci* genotypes with *C. abortus* S26/3 as an outgroup.

Annotated *pmp* and tbl3SS genes described by Voigt *et al.* (2012) from the 6BC genome (CP002549) were compared by blast analysis to the genomes sequenced in this study and six reference genomes available in GenBank [WC (NC_018624.1), M56 (NC_018623.1), VS225 (NC_018621.1), WS-RT-E3 (NC_018622.1), GR9 (NC_018620.1), RTH (SRA061571)] (Read *et al.*, 2013; Van Lent *et al.*, 2012; Voigt *et al.*, 2012). A formatted report was generated using an in-house gene search reporter pipeline (blast-based). The gene sequences that reported a pairwise identity below 75 % were marked as low quality and were considered absent.

A heatmap was constructed using R version 3.0.1 using the ‘gplots’ and ‘RColorBrewer’ packages (Neuwirth, 2007; Warnes et al., 2014). The *pmp* genes pairwise identities between all *C. psittaci* genomes included this study against *C. psittaci* 6BC were shown.

A maximum-likelihood tree was reconstructed with the *pmp* genes to show a similar evolutionary relationship in Voigt *et al.* (2012). We selected genes based on a protein pairwise identity to 6BC that met the following criteria: 75 % sequence identity and above 60 % gene coverage. ClustalOmega was used to perform independent multiple sequence alignments before sequence analysis to minimize gene rearrangement (Sievers *et al.*, 2011). Once each individual protein alignment was built, the independent alignments were concatenated. RAxML was used to generate a phylogenetic tree with 1000 replicates for bootstrapping (Stamatakis, 2014). The tree was visualized with Figtree (Andrew Rambaut, 2014).

### Protein domain identification

The gene sequences for CPSIT_0757 (dihydrodipicolinate reductase) and CPSIT_0192 (putative *TARP)* for 6BC genome (CP002549) were used as reference sequences to identify the orthologous gene sequences in the genomes included in this study. These three genes were selected based upon their critical role in virulence or metabolism and genetic variability between strains (Voigt *et al.*, 2012). Orthologous gene sequences were identified with a shared sequence identity greater than 75 %. Genomes GR9 (NC_0186201.1), M56 (NC_018623.1) and WS-RT-E30 (NC_018622.1) did not contain genes that met the criteria, thus excluding them from the protein domain identification analysis. The amino acid sequences for each gene were submitted to the European Bioinformatics Institute InterPro web service (Jones *et al.*, 2014). InterPro provides a functional analysis of protein sequences by predicting protein domains based on domain signatures found other protein family and domain databases (Jones *et al.*, 2014). A detailed description of the databases that make up InterProScan is given by Hunter *et al.* (2012).

## Results

### Whole genome sequencing, assembly and annotation

Table 1 summarizes the sequencing statistics and genomic characteristics for each newly sequenced genome included in this study. The sequencing resulted in assemblies covering 99 % or greater of the entire genome for each strain. All five strains yielded an approximately 1.16 Mb chromosome and a fully sequenced 7.5 kb plasmid. The plasmids were remarkably conserved with a pairwise identity of 99 %. The DNA G+C content for all five genomes was approximately 39 %, which is consistent with other sequenced *Chlamydia* genomes (Read *et al.*, 2013; Van Lent *et al.*, 2012). The genome coverage ranged from 340.4X (NJ1) to 581.1X (Frances), with a mean read length for all five genomes of 101 bp, and total reads generated were between 7 803 252 (NJ1) and 13 291 438 (Frances). The N50 values were between 775 399 (NJ1) and 1 159 687 (Frances). The numbers of genes predicted for each genome were similar. The lowest number of genes (1044) was predicted in the Frances genome compared to a high of 1059 genes in UGA. A total of 38 tRNAs were found in all five genomes.

### Pan genome comparison

**Fig. 1.**
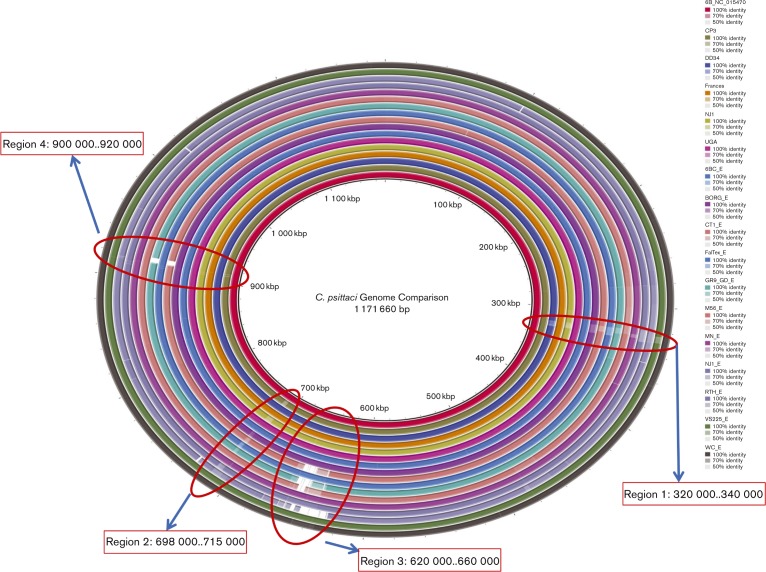
brig analysis. A brig comparing the *C. psittaci* genomes sequenced in this study and *C. psittaci* genomes sequenced by Read *et al.* (2013). Genotype 6BC was used as a reference for comparison. The innermost ring represents the base position along the map. The second ring indicates the GC content along the length of the genome. The colour rings correspond to the genomes with each genome indicated by a unique colour as indicated in the figure legend. A change in colour corresponds to a decrease in pairwise identity compared to the reference genome. Four regions with sequence divergence are noted in red circles with gene identities listed in orange boxes.

The 6BC strain was chosen as the reference with which to compare all other strains used in this study, because it was the only completely sequenced genome available at the start of this study. Fig. 1 is a circular map representing the nucleic acid sequence similarity of the five strains sequenced for this study and the genomes sequenced by Read *et al.* (2013) compared with the 6BC reference genome generated using the brig. The comparison shows a high degree of similarity ( ≥ 99 %) across all genotypes of *C. psittaci*. As expected, nearly identical sequence similarity was observed between the three genotype A genomes (6BC, UGA and DD34). While all the genomes appear to be very closely related, two regions demonstrated significant sequence divergence among the different genotypes. These regions are highlighted, and the genes encoded in each region are listed (Table S1, available in the online Supplementary Material). Region 1 contains approximately 20 kb; region 2 spans 17 kb. The majority of the genes contained in these regions encode Pmps belonging to the *pmp*G group. Several of the genomes sequenced by Read *et al.* (2013) had large deletions when compared with the reference 6BC.

**Fig. 2.**
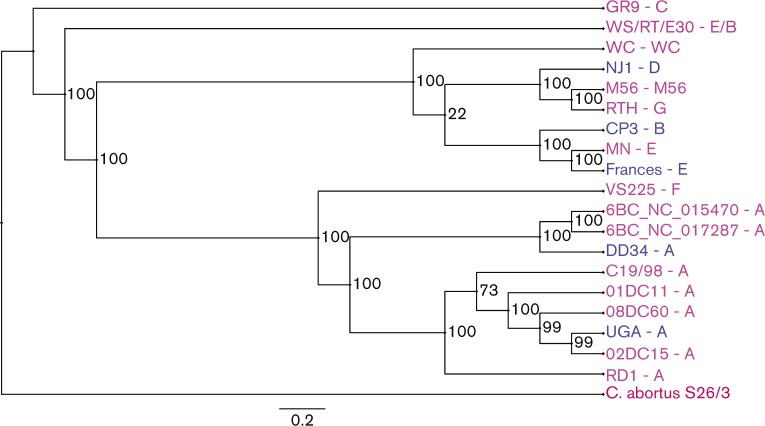
Whole genome SNP tree. A whole genome SNP tree rooted to *C. abortus* strain S26/3 reconstructed with all available genome sequences in the NCBI database along with the five genomes sequenced for this study. The strain name is followed by the genotype for each branch of the tree (Strain name–Genotype). Strain names colored in blue were sequenced for this study, names colored in pink were obtained from NCBI, and the genome colored in red was the root genome.

A whole genome SNP tree rooted to *C. abortus* strain S26/3 was reconstructed using previously reported *C. psittaci* genomes currently in the NCBI database and the genomes sequenced for this project (Fig. 2). The tree demonstrates the closely related nature of the *C. psittaci* genotypes. Nine genotype A strains are represented in the tree and formed the first clade. Genotype F was the next closest relative to genotype A, followed by the WC clade. A fourth clade composed of genotypes C and WS/RT/E30 was present, followed by genotypes B and E representing the fifth clade. Genotypes D, M56 and RTH formed their own separate branches, with strain RTH, genotype G, the most distantly related.

### T3SS and effector genes

**Fig. 3.**
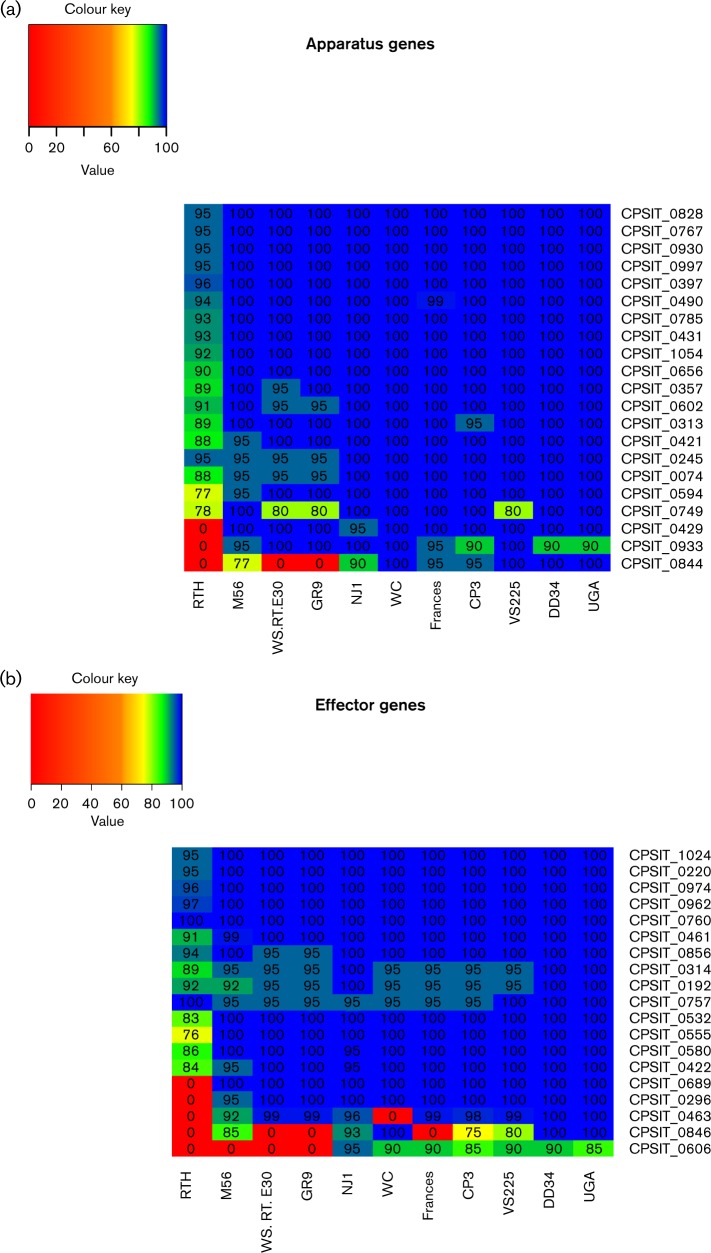
Heatmap analysis of tbl3SS genes. The nucleotide pairwise identities for (a) the apparatus genes and (b) the effector genes are shown for the strains in this study compared with 6BC tbl3SS reference genes. Pairwise identities of ≥ 75 % are shown. Pairwise identifies are coded with the following colour schema: red: 0–59 %, orange: 60 − 69 %, yellow: 70–79 %, green: 80–89 % and blue: 90–100 %.

Table 2 is a summary of the pairwise identity values ( ≥ 75 %) of the tbl3SS apparatus, chaperone and effector genes (*n* = 39) among all *C. psittaci* genotypes using the 6BC genome from Voigt *et al.* (2012) as the reference strain. There is a large degree of conservation amongst the genes comprising the tbl3SS. However, the effector genes show greater sequence divergence compared with the apparatus genes (Fig. 3a, b). Strain RTH (genotype G) was the most divergent, with only two genes sharing 100 % pairwise identity with 6BC. A large number of genes (16 out of 39) shared 100 % pairwise identity compared to 6BC for nine of the ten genotypes, with the RTH (genotype G) being the only divergent strain. However, substantial differences do exist among many of the genes. Two genes (CPSIT_0844 and CPSIT_0846) were absent in three or four strains (Table 2, yellow highlight). CPSIT_0844 and CPSIT_0846 encode IncA family proteins and were absent in GR9 (genotype C), RTH (genotype G) and WS/RT/E30 (genotype E/B); CPSIT_0846 was also absent in Frances (genotype E). A putative inner-membrane protein (CPSIT_0463) was absent in RTH and WC (genotypes G and WC, respectively).

**Table 2:** T3SS genes

		Strain:	DD34	UGA	CP3	GR9	NJ1	Frances	VS225	RTH	WS/RT/E30	M56	WC
		Genotype:	A	A	B	C	D	E	F	G	E/B	M56	WC
6BC locus tag (CP002549)	Name	Pairwise identity (%)											
**Apparatus genes**													
CPSIT_0074	Hypothetical protein		100	100	100	>95	100	100	100	88	>95	>95	100
CPSIT_0245	Cabohydrate isomerase		100	100	100	>95	100	100	100	95	>95	>95	100
CPSIT_0313	Polymorphic membrane protein, G family		100	100	>95	100	100	100	100	89	100	100	100
CPSIT_0357	Hypothetical protein		100	100	100	100	100	100	100	89	>95	100	100
CPSIT_0397	Hypothetical protein		100	100	100	100	100	100	100	96	100	100	100
CPSIT_0421	Hypothetical protein		100	100	10	100	100	100	100	88	100	>95	100
CPSIT_0429	Hypothetical protein		100	100	100	100	>95	100	100	–	100	100	100
CPSIT_0431	Putative membrane protein		100	100	100	100	100	100	100	93	100	100	100
CPSIT_0490	Hypothetical serine rich protein		100	100	100	100	100	>99	100	94	100	100	100
CPSIT_0594	IncA		100	100	100	100	100	100	100	77	100	>95	100
CPSIT_0602	Hypothetical protein		100	100	100	>95	100	100	100	91	>95	100	100
CPSIT_0656	Putative integral membrane protein		100	100	100	100	100	100	100	90	100	100	100
CPSIT_0749	Hypothetical protein		100	100	100	>80	100	100	>80	78	>80	100	100
CPSIT_0767	3-phosphoshikimate-1-carboxyvinyltransferase		100	100	100	100	100	100	100	95	100	100	100
CPSIT_0785	Hypothetical serine rich protein		100	100	100	100	100	100	100	93	100	100	100
CPSIT_0828	DNA recombination protein		100	100	100	100	100	100	100	95	100	100	100
CPSIT_0844	IncA family protein		100	100	>95	–	90	>95	100	–	–	>77	100
CPSIT_0930	tRNA (Uracil-5-)-methyltransferase		100	100	100	100	100	100	100	95	100	100	100
CPSIT_0933	Putative membrane protein		>90	>90	>90	100	100	>95	100	–	100	>95	100
CPSIT_0997	Putative inner membrane protein		100	100	100	100	100	100	100	95	100	100	100
CPSIT_1054	5-formyltetrahydrofolate-cyclo-ligase		100	100	100	100	100	100	100	92	100	100	100
**Effector genes**													
CPSIT_0192	tarp		100	100	>95	>95	100	>95	>95	92	>95	>92	>95
CPSIT_0220	Cyclodiphosphate synthase		100	100	100	100	100	100	100	95	100	100	100
CPSIT_0296	Hypothetical protein		100	100	100	100	100	100	100	–	100	>95	100
CPSIT_0314	Polymorphic membrane protein, G family		100	100	>95	>95	100	>95	>95	89	>95	>95	>95
CPSIT_0422	Hypothetical protein		100	100	100	100	>95	100	100	84	100	>95	100
CPSIT_0461	Hypothetical protein		100	100	100	100	100	100	100	91	100	99	100
CPSIT_0463	Putative inner-membrane protein		100	100	>98	99	96	>99	99	–	99	>92	–
CPSIT_0532	IncB		100	100	100	100	100	100	100	83	100	100	100
CPSIT_0555	Putative inner-membrane protein		100	100	100	100	100	100	100	76	100	100	100
CPSIT_0580	Putative inner-membrane protein		100	100	100	100	>95	100	100	86	100	100	100
CPSIT_0689	Hypothetical protein		100	100	100	100	100	100	100	–	100	100	100
CPSIT_0757	Dihydrodipicolinate reductase		100	100	>95	>95	>95	>95	100	100	>95	>95	>95
CPSIT_0760	Hypothetical membrane protein		100	100	100	100	100	100	100	100	100	100	100
CPSIT_0846	IncA family protein		100	100	>75	–	>93	–	>80	–	–	>85	100
CPSIT_0856	Membrane protein		100	100	100	>95	100	100	100	94	>95	100	100
CPSIT_0962	Flagellar biosynthesis/type III secretory pathway		100	100	100	100	100	100	100	97	100	100	100
CPSIT_0974	Trigger factor		100	100	100	100	100	100	100	96	100	100	100
CPSIT_1042	Deoxyribonucleotide triphosphate pyrophosphatase		100	100	100	100	100	100	100	95	100	100	100

Locus tags highlighted in yellow are absent in one or more strains, and those highlighted in blue are discussed in the manuscript. –, The gene was not present.

The effector protein dihydrodipicolinate reductase (CPSIT_0757) was conserved among DD34 and UGA (genotypes A), VS225 (genotype F) and RTH (genotype G) but displayed differences in the other seven genotypes (Table 2, blue highlight). While the InterPro analysis revealed two domains for a NAD(P) binding domain present in all genotypes, a multiple sequence alignment of those domains showed that DD34, UGA, VS 225 and RTH clustered together (Fig. S1, magenta highlight) and WS/RT/E30, GR9, WC, Frances, CP3, M56 and NJ1 formed a second cluster (Fig. S1, underlined). In the first domain, eight of ten substitutions were clustered in this fashion, while the second domain showed nine of thirteen substitutions sharing this pattern. Other sporadic changes within the catalytic domains were also observed requiring further study. M56 had a single substitution compared to the other strains in the first domain (Fig. S1, blue highlight), and NJ1 had a unique substitution in the C-terminal domain (Fig. S1, red highlight).

Other effector genes such as the *tarp* gene (CPSIT_0192) were 100 % conserved among DD34, UGA and NJ1 (genotypes A and D, respectively), but there were differences in the other strains, as the pairwise identity was 92 % or greater in each. To identify functional regions of the *tarp* gene, a nucleotide and amino acid sequence alignment (Fig. S2, S3) were performed using the genomes sequenced in this study, those sequenced by Read *et al.* (2013), and *C. trachomatis* L_2_ and L_2_b strains (accession numbers AM884176 and AM884177) characterized by Somboonna *et al.* (2011). The *tarp* genes in the *C. psittaci* genomes had very little sequence similarity with the two *C. trachomatis* genomes (Fig. S2). The *C. psittaci* amino acid sequences of Tarp were examined in InterPro and three catalytic domains of unknown function were returned that were present in all strains. A multiple sequence alignment of those domains revealed five amino acid differences in each of the three domains (Fig. S3, green highlight). Of these fifteen substitutions, four were specific to the RTH strain, two were only present in the M56 strain, eight were shared between the M56 and RTH strains and one amino acid substitution was only present in the NJ1 strain (Fig. S3, green highlight).

The IncA (CPSIT_0594) and IncB (CPSIT_0532) were largely conserved. The *incA* gene was 100 % conserved in every genome with the exception of RTH and M56, whereby the pairwise identity was 77 % and >95 % respectively. The *incB* gene was 100 % conserved in 10 genomes compared to 6BC, with RTH being the only exception with a pairwise identity of 83 %.

### Pmp genes

**Fig. 4.**
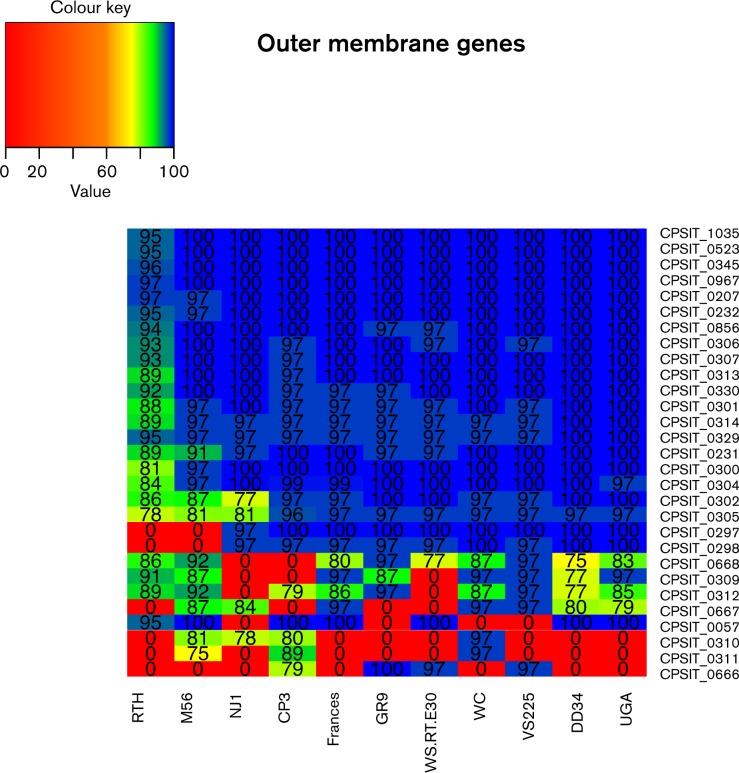
Heatmap analysis of Pmp genes. The nucleotide pairwise identities for all 30 *C. psittaci* Pmp genes are shown for the strains in this study compared with 6BC Pmp reference genes. Pairwise identities of ≥ 75 % are shown. Pairwise identifies are coded with the following colour schema: red: 0–59 %, orange: 60 − 69 %, yellow: 70–79 %, green: 80–89 % and blue: 90–100 %.

The 30 Pmps characterized by Voigt *et al.* (2012) were compared with the strains sequenced in this study as well as the other strains with whole genome data now available (Table 3). The BRIG analysis (Fig. 1, regions 1 and 2) revealed that the G group of proteins had the highest degree of divergence among the different genotypes of *C. psittaci*. Fig. 4 shows the heatmap of the Pmps showing that the *pmp*G group is clearly the most diverged. Of the 14 genes of this group, only seven were present in all 12 genomes. Also notable is that, among the three genotype A strains (6BC, DD34 and UGA), three genes in the G family were present only in the 6BC strain. Two genes (CPSIT_0310 and CPSIT_0311) were only present in 6BC, CP3, M56 and WC, and a weak match was also returned for CPSIT_0310 in NJ1. Strain 6BC and WC had the largest conservation of this group of genes. We found 13 of the 14 *pmpG* genes in the 6BC had significant pairwise identity (>85 %) with those in WC.

**Table 3:** Polymorphic outer-membrane protein genes

PMP family	6 BC locus tag (CP002549)	Strain:	DD34	UGA	CP3	GR9	NJ1	Frances	VS225	RTH	WS/RT/E30	M56	WC
		Genotype:	A	A	B	C	D	E	F	G	E/B	M56	WC
		Pairwise identity (%)											
A	CPSIT_0232		100	100	100	100	100	100	100	95	100	>95	100
B/C	CPSIT_0231		100	100	100	>95	>95	100	100	89	>95	>90	100
D	CPSIT_0856		100	100	100	>95	100	100	100	94	>95	100	100
E/F	CPSIT_0297		100	100	100	100	>95	100	100	–	100	–	100
	CPSIT_0298		100	100	>95	>95	>95	>95	>95	–	>95	–	100
G	CPSIT_0302		100	100	>95	100	>75	>95	>95	86	100	>84	>95
	CPSIT_0304		100	>95	>99	100	100	>99	100	84	100	>95	100
	CPSIT_0305		>95	>95	>94	>95	81	>95	>95	78	>95	>79	>95
	CPSIT_0306		100	100	>95	100	100	100	>95	93	>95	100	100
	CPSIT_0307		100	100	>95	100	100	100	100	93	100	100	100
	CPSIT_0309		>75	>96	–	>85	–	>95	>95	91	–	>85	>95
	CPSIT_0310		–	–	80	–	>76	–	–	–	–	>79	>95
	CPSIT_0311		–	–	>87	–	–	–	–	–	–	75	>95
	CPSIT_0312		>76	>83	>76	>95	–	>84	>95	89	–	>90	>85
	CPSIT_0313		100	100	>95	100	100	100	100	89	100	100	100
	CPSIT_0314		100	100	>95	>95	>95	>95	>95	89	>95	>95	>95
	CPSIT_0666		–	–	>77	100	–	–	>95	–	>95	–	–
	CPSIT_0667		>78	>78	–	–	84	>95	>95	–	–	>85	>95
	CPSIT_0668		75	>81	–	>95	–	80	>95	86	>75	>90	>85
H	CPSIT_0301		100	100	>95	>95	100	>95	>95	88	>95	>95	100
Other	CPSIT_0057		100	100	100	–	–	100	–	95	100	100	–
	CPSIT_0207		100	100	100	100	100	100	100	97	100	>95	100
	CPSIT_0300		100	100	100	100	100	100	100	81	10	>95	100
	CPSIT_0329		100	100	>95	>95	>95	>95	>95	95	>95	>95	>95
	CPSIT_0330		100	100	>95	>95	100	>95	100	92	100	100	100
	CPSIT_0345		100	100	100	100	100	100	100	96	100	100	100
	CPSIT_0523		100	100	100	100	100	100	100	95	100	100	100
	CPSIT_0967		100	100	100	100	100	100	100	97	100	100	100
	CPSIT_1035		100	100	100	100	100	100	100	95	100	100	100

“–” indicates the gene was not present.

The other Pmp gene groups had significantly higher conservation among the genotypes. Genes of the *pmpA*, *pmp*B/C, *pmp*D and *pmp*H groups were present in all 12 genomes, with a small degree of sequence divergence present among some of the genes. The most divergent gene was CPSIT_0231 of the *pmp*B/C family. Its corresponding gene in the M56 genome had a 90 % pairwise identity and an 89 % identity in the RTH genome. All the other pairwise identities for those families were 88 % or greater among all 12 genomes. The *pmp*E/F genes were the only ones that were absent in any of the genomes. Both genes were not present in the RTH and M56 genomes. A heat map demonstrates the variability of the *pmp*G group of genes among the strains compared to the other Pmp groups (Fig. 4).

**Fig. 5.**
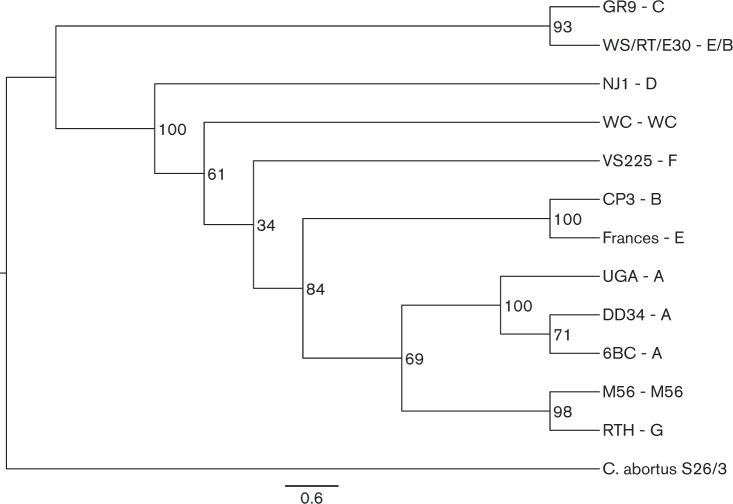
Phylogenetic tree of Pmp genes. Phylogenetic relationships computed using the maximum-likelihood from the conserved Pmp genes found in the *C. psittaci* genomes included in this study. Protein sequences with a pairwise identity ≥ 75 % to the *C. psittaci* 6BC genome were included. Bootstrapping percentage values are shown at branch points, and the bar represents the residue substitution per site. The strain name is followed by the genotype for each branch of the tree (Strain name–Genotype).

In reconstructing a phylogenetic tree of the Pmps for each strain with Pmps, the phylogeny is similar to the whole-genome phylogeny of Fig. 2 (Fig. 5).

## Discussion

*Chlamydiaceae* has one of the largest host ranges and varied virulence characteristics of any bacterial family currently known (Kaleta & Taday, 2003). Application of next-generation sequencing technologies has yielded novel information about *C. psittaci* genomes and provided insight into the differing virulence mechanisms, tissue tropism, evolutionary dynamics and host range of this species (Read *et al.*, 2013; Voigt *et al.*, 2012). Voigt *et al.* (2012) compared the genes of other *Chlamydia* species with hose of *C. psittaci* strain 6BC and found a number of *psittaci*-specific Pmps of the G family, a high degree of genome rearrangement, and numerous differences in the tbl3SS. Read *et al.* (2013) demonstrated *C. psittaci* has undergone many recombination events and has the ability to switch hosts frequently. Our phylogenetic analysis revealed similar genotypic clade relationships seen by Read *et al.* (2013) and Voigt *et al.* (2012). The current study further enhances our understanding of this species by identifying a variety of genetic variations among the *pmp* and the tbl3SS genes that may, in part, be responsible for the variability in tissue tropism, host preferences, and virulence observed among genotypes.

*Chlamydiaceae* contains a functional tbl3SS that serves as a major virulence factor by secreting effectors that recruit actin to facilitate entry into the cell (TARP), manipulate the host cell cytoplasm (CdsF), and alter the inclusion body (IncG and IncA) (Betts *et al.*, 2008; Fields *et al.*, 2003; Hsia *et al.*, 1997; Voigt *et al.*, 2012). Studies have identified numerous apparatus proteins along with multiple chaperone proteins and secreted effector molecules (Betts *et al.*, 2008; Herrmann *et al.*, 2006; Peters *et al.*, 2007; Stone *et al.*, 2008; Voigt *et al.*, 2012). The type III apparatus proteins are highly conserved among many bacterial species including *Shigella*, *Yersinia* and *Salmonella* (Hueck, 1998; Mota & Cornelis, 2005). This conservation is so extensive that small molecule inhibitors such as INP0400 designed for one species have efficacy against chlamydial species secretion systems, including IncG and IncA, and can inhibit the development of C*hlamydia* after entry into host cells (Muschiol *et al.*, 2009).

Intraspecies comparisons of the tbl3SS genes (Voigt *et al.*, 2012) showed that the majority of the genes were largely conserved in all 12 *C. psittaci* genomes with a pairwise identity of 95 %. However, we found significant differences for some genes among the different genotypes of *C. psittaci*. For example, seven of the 39 genes were missing in one or more of the genotypes compared with 6BC (Table 2, yellow highlight). Of these seven, three are hypothetical proteins, two are associated with the IncA protein family, and two are putative membrane proteins. For the other 31 genes, there was little sequence divergence with pairwise identities ranging from 77 % to 100 %. However, all apparatus structure genes had at least a 95 % pairwise identity except for the RTH strain (genotype G) where the identities were more variable.

Differences in the TARP, dihydrodipicolinate reductase and the adherence factor genes appear to impact virulence and host specific characteristics within the different *C. psittaci* genotypes. *tarp* was less conserved among the strains, which is consistent with a report showing that virulent strains of *C. psittaci* such as genotypes A and D recruit actin more efficiently than those associated with less virulence strains (Beeckman & Vanrompay, 2010). The increase in actin recruitment may provide a more efficient mechanism for EB attachment, invasion and inclusion formation. The amino acid sequences of the TARP suggest that this particular gene may be responsible for differences in the M56 (genotype M56) and RTH (genotype G) strains as the majority of the substitutions in the catalytic domains were associated with those two strains. This is not surprising as these two strains have widely different host preferences compared to the other genotypes. Further experiments are needed to better elucidate the functional properties of the *C. psittaci tarp* gene and protein among the different strains and genotypes, and to identify other possible genes involved in virulence.

Sequence divergence and amino acid changes in the dihydrodipicolinate reductase gene (Cpsit_0757) suggest a potential difference in lysine biosynthesis for 6BC, DD34 and UGA (genotypes A), VS225 (genotype F) and RTH (genotype G) compared with the other *C. psittaci* genotypes. These substitutions could have important consequences for cell wall synthesis as lysine has been shown to be an important amino acid in the synthesis of peptidoglycan in other bacteria (Pavelka & Jacobs, 1996). This, combined with the recent report providing strong evidence for the presence of peptidoglycan in the cell wall of *Chlamydia*, warrants further investigation of this protein (Liechti *et al.*, 2014).

The *incA* (CPSIT_0594) and *incB* (CPSIT_0532) genes were remarkably conserved among all the genotypes displaying 100 % pairwise identity, except for the RTH strain (genotype G) that had 77 % and 83 % pairwise identities, respectively. These genes were highly variable among the different species within the *Chlamydiaceae* family, but this divergence is not observed past the species level (Voigt *et al.*, 2012). These data suggest these genes operate in the same manner among the majority of *C. psittaci* strains, but further *in vitro* studies will help to determine how their function compares to their orthologues in the RTH strain and other *Chlamydia* species.

The Pmps are another major source of diversity among the different species of the *Chlamydiaceae* family as shown for *C. abortus* and *C. psittaci* in prior studies (Thomson *et al.*, 2005; Voigt *et al.*, 2012). We compared the Pmps identified by Voigt *et al.* (2012) in addition to nine genes identified as membrane proteins from our annotation analysis. Among the different subsets of outer-membrane protein genes, the *pmp*G group has been demonstrated to be the most divergent. This family of proteins has previously been described as the most rapidly evolving group of proteins, exhibiting numerous deletion and duplication events among other *Chlamydiaceae* (Thomson *et al.*, 2005). Voigt *et al.* (2012) characterized several new *pmpG* genes in *C. psittaci* that were not present in any other *Chlamydia* species. This study was able to conclusively determine that this diversity extends to the different genotypes of *C. psittaci*. The number of Pmps missing in some genomes, and the large sequence divergence observed among strains compared to 6BC, even within the same genotype A, suggests that these genes may be used to rapidly adapt to different environments (Fig. 4). While some sequence divergence was seen among other species, it is possible that the *pmp*G group plays a major role in tissue tropism and host preferences of the different strains of *C. psittaci* because of their diverse nature and ability to rapidly evolve (Read *et al.*, 2013; Voigt *et al.*, 2012). The Pmp tree (Fig. 5) is similar to the whole genome phylogeny suggesting that these genes may significantly contribute to strain and genotype diversity. A follow up study sequencing a number of different strains of the same genotype from diverse geographical regions and animal species would be helpful in determining if this family of genes is responsible for phenotypic differences. This will also allow a comprehensive evaluation to determine if variant genotypes are emerging due to the rapidly evolving nature of Pmps.
